# Improved Estimation of Cardiac Function Parameters Using a Combination of Independent Automated Segmentation Results in Cardiovascular Magnetic Resonance Imaging

**DOI:** 10.1371/journal.pone.0135715

**Published:** 2015-08-19

**Authors:** Jessica Lebenberg, Alain Lalande, Patrick Clarysse, Irene Buvat, Christopher Casta, Alexandre Cochet, Constantin Constantinidès, Jean Cousty, Alain de Cesare, Stephanie Jehan-Besson, Muriel Lefort, Laurent Najman, Elodie Roullot, Laurent Sarry, Christophe Tilmant, Frederique Frouin, Mireille Garreau

**Affiliations:** 1 Laboratoire d’Imagerie Biomédicale, Institut National de la Santé et de la Recherche Médicale, Centre National de la Recherche Scientifique, Université Pierre et Marie Curie, Paris, France; 2 École Spéciale de Mécanique et d’Électricité-Sudria, Ivry-sur-Seine, France; 3 Laboratoire Electronique, Informatique et Image, Centre National de la Recherche Scientifique, Université de Bourgogne, Dijon, France; 4 Centre de Recherche en Acquisition et Traitement de l’Image pour la Santé, Centre National de la Recherche Scientifique, Institut National de la Santé et de la Recherche Médicale, Institut National des Sciences Appliquées Lyon, Université de Lyon, Villeurbanne, France; 5 Unité d’Imagerie Moléculaire In Vivo, Service Hospitalier Frédéric Joliot, Institut National de la Santé et de la Recherche Médicale, Centre National de la Recherche Scientifique, Commissariat à l’Energie Atomique, Université Paris Sud, Orsay, France; 6 Laboratoire d’Informatique Gaspard Monge, Centre National de la Recherche Scientifique, Université Paris-Est Marne-la-Vallée, École Supérieure d’Ingénieurs en Électrotechnique et Électronique, Marne-la-Vallée, France; 7 Groupe de Recherche en Informatique, Image, Automatique et Instrumentation de Caen, Centre National de la Recherche Scientifique, Caen, France; 8 Image Science for Interventional Techniques, Centre National de la Recherche Scientifique, Université d’Auvergne, Clermont-Ferrand, France; 9 Institut Pascal, Centre National de la Recherche Scientifique, Université Blaise Pascal, Clermont-Ferrand, France; 10 Laboratoire de Traitement du Signal et des Images, Institut National de la Santé et de la Recherche Médicale, Université de Rennes, Rennes, France; Scuola Superiore Sant'Anna, ITALY

## Abstract

This work aimed at combining different segmentation approaches to produce a robust and accurate segmentation result. Three to five segmentation results of the left ventricle were combined using the STAPLE algorithm and the reliability of the resulting segmentation was evaluated in comparison with the result of each individual segmentation method. This comparison was performed using a supervised approach based on a reference method. Then, we used an unsupervised statistical evaluation, the extended Regression Without Truth (eRWT) that ranks different methods according to their accuracy in estimating a specific biomarker in a population. The segmentation accuracy was evaluated by estimating six cardiac function parameters resulting from the left ventricle contour delineation using a public cardiac cine MRI database. Eight different segmentation methods, including three expert delineations and five automated methods, were considered, and sixteen combinations of the automated methods using STAPLE were investigated. The supervised and unsupervised evaluations demonstrated that in most cases, STAPLE results provided better estimates than individual automated segmentation methods. Overall, combining different automated segmentation methods improved the reliability of the segmentation result compared to that obtained using an individual method and could achieve the accuracy of an expert.

## Introduction

Cardiac Magnetic Resonance Imaging (cMRI) is used more and more frequently in clinical routine to study simultaneously the cardiac anatomy and function. A series of clinical parameters can be deduced from the acquired scans in cMRI. Among these parameters, the left ventricular ejection fraction (LVEF) remains a major prognostic index for coronary artery diseases assessment. The correct estimation of this parameter requires the accurate measurement of both end-diastolic volumes (*EDV*) and end-systolic volumes (*ESV*), providing the stroke volume (*SV*) and finally the LVEF. In addition, the proper delineation of epicardial border providing the epicardial volume (*EpV*) is also necessary to estimate the myocardial mass (*MM*). Although MRI makes these measurements possible with a high accuracy (generally from a series of short-axis cine-MR images), the segmentation of the left ventricle (LV) is still a contemporary issue [1] due to the considerable amount of data that are acquired in a single examination. For clinical routine, semi-automated algorithms that are proposed by commercial image post-processing software are largely used. For retrospective studies, research studies, or large database studies, automated segmentation algorithms are preferentially used in order to avoid the labor intensive and time consuming manual segmentation task and reduce the intra- and inter-operator variabilities [2]. To assess the performance of these automated segmentation algorithms, the common approach consists in comparing the contours resulting from the automated segmentation with the ones obtained by one or several experts who are known to often outperform automated methods [3].

When visually comparing segmentation results obtained by different automated methods as in [3], the respective performance of two methods depends on the data: when a first segmentation method provides more accurate contours than a second method on a specific database, the second algorithm might actually be more relevant for a sub-database or, at least, for some particular MR examinations. Therefore, it is reasonable to hypothesize that there might be an advantage in combining several automated segmentation methods to overcome the specific limitations of each one.

To combine segmentation approaches, different algorithms have been proposed [4–8]. The Simultaneous Truth and Performance Level Estimation (STAPLE) algorithm [5] is very popular and highly cited. Furthermore, the associated software is freely available for academic purposes upon written request. For these reasons, we evaluated the performance of STAPLE. To objectively assess the segmentation accuracy, criteria based on estimated contours and associated image classification are often used. These include various metrics allowing to compare boundaries at a local level such as distances between contours, overlap criteria like the Dice coefficient [9], or the sensitivity, the specificity, the predictive negative value and the predictive positive value criteria computed by the STAPLE algorithm. All these criteria assume that there is a “gold standard” segmentation, at least implicitly. Furthermore, these criteria are partly correlated and are also directly related to the optimization process involved in STAPLE. To avoid these limitations, we rather focused our evaluation on the clinical task and evaluated the accuracy of clinical parameters of interest, and particularly the LVEF parameter.

To evaluate the interest of the STAPLE algorithm for combining segmentation results, we applied it to a cardiac cine MRI database including LV segmentation obtained from eight independent segmentation approaches: five resulted from five different automated image processing approaches, and three volume contours were drawn by three different experts. Sixteen combinations of the five automated methods (all five methods, four among the five methods, and three among the five methods) were tested against results provided by the three experts, using the LVEF values as the clinical parameter of interest. The evaluation was first carried out using a supervised approach, assuming a gold standard was available, and then using an unsupervised approach, the extended Regression Without Truth (eRWT) [3] to rank all segmentation methods as a function of their performance.

Our study presents some similarities with [2]: both used a public cardiac cMRI database (although not the same) for which contours were delineated by experts and algorithms. In our case, the selected database included controls and patients with different cardiac pathologies. In [2], only cMRI acquired on patients were included. Furthermore, both studies used STAPLE to combine different contour results, but they differ in their approach. Indeed, in [2], authors proposed to use STAPLE to define a gold standard segmentation based on two fully-automated algorithms and three semi-automated algorithms requiring manual input, while the present study focuses on improving the accuracy of automated segmentation algorithms by combining them with STAPLE to get a accuracy similar to the one achieved by experts *i.e.* make it acceptable for clinical routine. To complete our first study [3] that enabled us to rank expert delinations and automated segmentation methods on the Cardiac MR Left Ventricular Segmentation Grand Challenge (MICCAI 2009) database [10], the present study aimed at demonstrating, using the same database, the usefulness of combining the different automated approaches that were previously independently evaluated.

## Materials and Methods

### Database

This work uses the public database provided by Sunnybrook Health Sciences Center [10]. This cardiac database was first distributed to the participants in the Cardiac MR Left Ventricular Segmentation Grand Challenge (MICCAI 2009). It includes images from forty-five subjects who were divided into four subgroups: healthy individuals (CTRL, n = 9), patients with hypertrophic cardiomyopathy (HYP, n = 12), patients with heart failure without ischemia (HF-NI, n = 12) and patients with heart failure due to ischemia (HF-I, n = 12). For each examination, about ten short axis slices covering the LV were acquired using a breath-hold, retrospective ECG-gated cine-MRI sequence (twenty cardiac phases per slice, contiguous slices with a slice thickness of 8 mm, FOV = 320 mm, acquisition matrix 256 × 256 with a 1.5T MR scanner (GE Healthcare)).

We focused here on the left ventricular ejection fraction (LVEF) estimate. LVEF was calculated conventionally as the ratio between the stroke volume and the end-diastolic volume. The end-diastolic and end-systolic volumes were measured from the endocardial border that was delineated on each selected image. MR images corresponding to the end-systolic and end-diastolic phases in the cardiac cycle as well as the list of consecutive slices considered for the LV segmentation were *a priori* fixed for this Challenge and given to the experts to directly compare results between all participants. The variability due to the choice of these temporal phases and of the associated slices was out of the scope of this study which focused on 2D slice segmentation.

### Segmentation approaches

Eight independent estimates of the LVEF were obtained from three manual contouring methods (*M*1-*M*3) provided by three independent experts, and from five automated algorithms (*M*4-*M*8). The five algorithms described respectively in [11–15] use different segmentation strategies and various user’s interactions. The method *M*8 was described in [15] and is an update of the method [16] previously evaluated in [3]. Endocardial borders were obtained on the end-diastolic and end-systolic phases with all methods. Furthermore, contours for all cardiac phases were provided for the whole database by method *M*5 and for fifteen subjects by method *M*6. All methods but *M*5 included the papillary muscles in the LV cavity. Method *M*4 was the least automated one, while method *M*8 was fully automated. Results obtained by the eight segmentation methods are freely available on https://github.com/frederiquefrouin/Medieval.

Using each segmentation method, the mean LVEF value and its associated standard deviation were calculated for each of the four subgroups of subjects. More than 99% of these estimated values ranged from 0.05 to 0.85. The twenty-four patients of the studied database with heart failure (HF-NI and HF-I) had a reduced LVEF that was considered as pathological (≤ 0.45).

### Combination of the segmentation approaches

#### Method

Several segmentation results were combined using the Simultaneous Truth and Performance Level Estimation (STAPLE) algorithm developped by Warfield *et al.* [5]. This method was implemented using the version 1.5.2 of CRKit, which is the software provided by Warfield’s team.

The STAPLE framework is based on an Expectation Maximization (EM) algorithm [17, 18]. It uses several segmentation results and calculates simultaneously a probabilistic estimate of a representative segmentation result and a performance level of each delineation included in the calculation. This performance level is provided by the computation of the sensitivity and the specificity indexes between each input segmentation and the segmentation result. The process is iterated until a stable solution is reached. Here, the STAPLE algorithm was run using the default parameters that were proposed by its authors. The binary version was used since only two classes were considered: the left ventricle and the remaining structures outside the left ventricle. Provided results did not depend on the size of the background (the region of interest surrounding the left cavity in our application) as mentioned in [2]. Furthermore, the STAPLE algorithm was applied in 2D, for each slice separately in order to be compliant with most of the initial segmentation methods, and because of the large slice thickness compared with the in-plane resolution. The resulting contours were stacked to get a 3D segmentation result.

#### Application

The STAPLE algorithm was applied to several combinations of endocardial segmentation results obtained from the five automated methods previously described:

- a STAPLE segmentation *MS*45678 was created from the five automated methods.
- STAPLE was used to combine all five combinations of four automated methods. For instance, the resulting segmentation was denoted *MS*4567 when methods *M*4, *M*5, *M*6 and *M*7 were involved in the algorithm.
- STAPLE was also applied to each combination of three automated methods among the five available (10 combinations). The result was denoted *MS*456 when methods *M*4, *M*5 and *M*6 were involved in the algorithm.

Using each STAPLE segmentation result, the mean LVEF value and associated standard deviation were calculated for each of the four subgroups of subjects.

### Supervised evaluation

In case of supervised evaluation, it is necessary to define a gold standard. For our problem of contour delineation on clinical data there is no ground truth reference, even when three experts have delineated contours [4]. We could have used STAPLE to define a consensus as proposed for instance in [2]. Yet, in order to be independent of STAPLE for the evaluation, we defined *M*2 as the reference method (*Mref*). Indeed, it was shown in [3] that method *M*2 performed the best and that the LVEF obtained by the three experts were more accurate than any of the five automated methods that were tested. The supervised evaluation was based on the computation of the bias *β* and its associated standard deviation (*s*) of each segmentation method *Mj* with respect to the reference *M*2, (*j* representing either one of the original methods or one of the sixteen STAPLE combinations described above).

### Unsupervised evaluation using eRWT

#### Theory

The eRWT approach [3], an extension of the Regression Without Truth [19–21], aims at comparing and ranking different methods which estimate a specific biomarker such as the LVEF, the true value Θ_*p*_ of the biomarker being unknown. Considering *P* samples (denoted by *p*, ranging from 1 to *P*) and *K* segmentation methods (denoted by *Mk*, *k* ranging from 1 to *K*), each segmentation method *Mk* yields an estimate *θ*_*pk*_ of the biomarker for sample *p*.

The eRWT approach assumes a parametric relationship between the true value Θ_*p*_ and its estimate *θ*_*pk*_ based on three hypotheses:

*H*1: The statistical distribution of the true value Θ_*p*_ on the whole database has a finite support.
*H*2: The estimate *θ*_*pk*_ is linearly related to the true value (Eq (1)). The error term *ɛ*_*pk*_ is normally distributed with zero mean and standard deviation *σ*_*k*_. The *a*_*k*_ and *b*_*k*_ parameters are specific to each method *Mk* and independent of sample *p*:
  $$\theta_{pk}=a_{k}\Theta_{p}+b_{k}+\varepsilon_{pk}.$$ (1)
*H*3: The error terms *ɛ*_*pk*_ for each method *Mk* are statistically independent.

With regard to *H*1, a Beta distribution *Beta*(*μ*, *ν*) was chosen for LVEF as it had been proposed in [19]. Besides, given all these assumptions, the probability of the estimated values *θ*_*pk*_ given the linear model parameters and the true value Θ_*p*_ can be expressed and the log-likelihood can be written as a function of *a*_*k*_, *b*_*k*_, *σ*_*k*_ and the probability distribution of Θ_*p*_.

The maximization of this log-likelihood does not require the numerical values of the true LVEF, but only a model of its statistical distribution (*pr* (Θ_*p*_)); it leads to the estimates of the linear model parameters for each method (*a*_*k*_, *b*_*k*_ and *σ*_*k*_).

The numerical implementation uses an optimization function implemented in MATLAB (R2012a, The Mathworks, Inc.). The figure of merit *F*_*Mk*_ chosen to rank the methods *Mk* is defined as the expected value of the square error between the true value of the parameter Θ_*p*_ and its estimated value by a given method (Eq (2)) [22].

$$F_{Mk}=𝔼[{\left(\Theta -a_{k}\Theta -b_{k}-\varepsilon_{k}\right)}^{2}].$$ (2)

If the statistical distribution of Θ_*p*_ is a Beta distribution, the figure of merit can be expressed analytically by Eq (3):

$$\begin{array}{ccc} F_{Mk} & = & {\left(a_{k}-1\right)}^{2}\frac{\mu (\mu +1)}{\left(\mu +\nu )(\mu +\nu +1\right)}+\\ & & 2(a_{k}-1)b_{k}\frac{\mu }{\mu +\nu }+b_{k}^{2}+\sigma_{k}^{2}. \end{array}$$ (3)

To set the shape parameters of the Beta distribution (*μ* and *ν*), we started from the values chosen in [3] (*μ* = 4 and *ν* = 5) and refined these initial values so as to minimize the sum of the *K* figures of merit. Final values of the *μ* and *ν* parameters were set to 2.85 and 3.40 respectively. These slight modifications of the Beta distribution compared to that used in [3] did not yield substantial changes in the ranking of the methods, as already shown in [3].

The final ranking of methods was based on a bootstrap process [23] running on the database of *P* values *θ*_*pk*_ generating *N* (*N* = 1000) *θ*_*p*^*n*^*k*_ values. From each drawing *n*, *P* values *p* ^*n*^ were drawn from the 45 samples. From these *θ*_*p*^*n*^*k*_ values, the *K* figures of merit $F_{Mk}^{n}$ were computed using the previously described optimization. The non-parametric Kruskal-Wallis test [24] was applied to the *N* × *K* values of $F_{Mk}^{n}$ to test the equality of the median among the *K* methods. When it was not equal, each pair of methods was tested, using a Bonferroni correction with a Type I error equal to 5% [25] to determine the significantly different pairs.

#### Experiments

The eRWT approach was first performed to rank the eight segmentation methods (*M*1 − *M*8). This ranking approach was then systematically applied to the eight methods *M*1 − *M*8 and to one of the STAPLE results to rank each segmentation combination, *MSi*, among the eight initial segmentation methods.

## Results

### Combination of the segmentation approaches

#### Superimposition of contours resulting from different segmentation methods on cMRI

**Fig 1:**
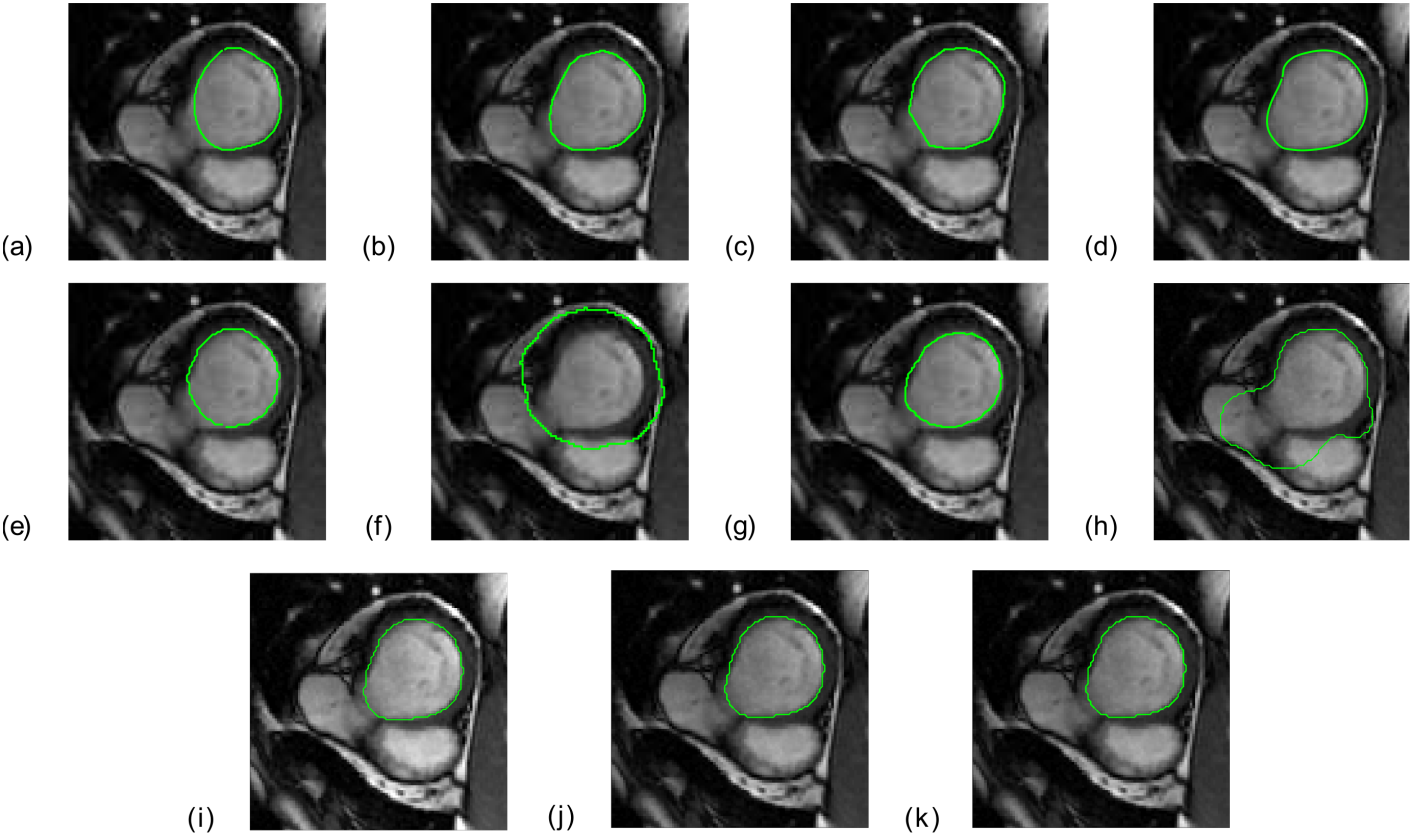
Basal cine MRI slice at end-diastole with superimposed contours of the LV (green line). *M*1 to *M*8 are represented from (a) to (h) and three different combinations of the STAPLE algorithm, *MS*45678, *MS*456 and *MS*4578 are represented from (i) to (k).

**Fig 2:**
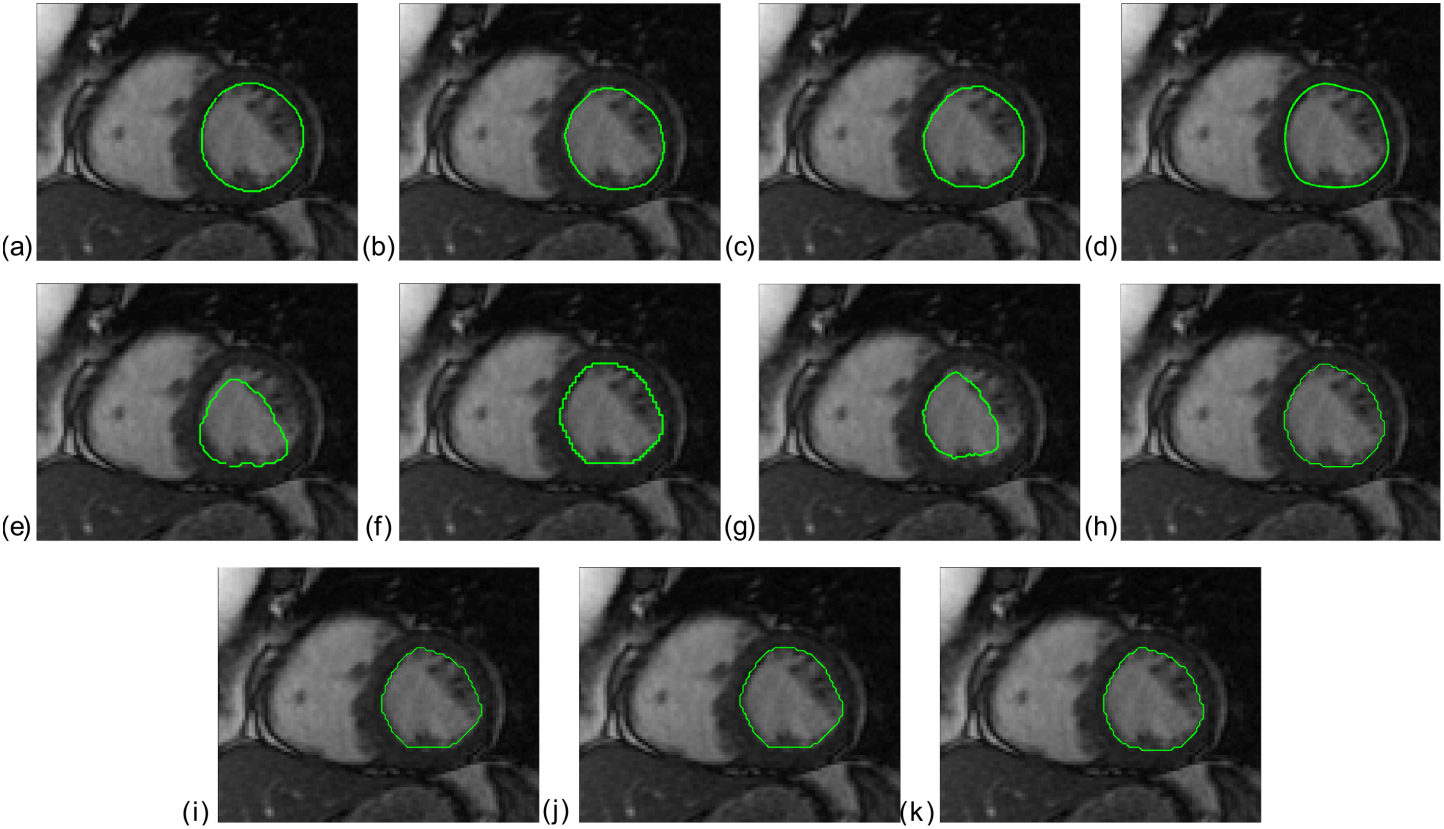
Median cine MRI slice at end-diastole with superimposed contours of the LV (green line). *M*1 to *M*8 are represented from (a) to (h) and three different combinations of the STAPLE algorithm, *MS*45678, *MS*456 and *MS*4578 are represented from (i) to (k).

Figs 1 and 2 show the endocardial contours obtained using the eight segmentation approaches *M*1 − *M*8 and using three different STAPLE combinations, superimposed on an end-diastolic image. These two figures correspond to two different cases: one patient (SC-HF-01) and one control (SC-N-05). In these two examples, the LV contour was correctly delineated by the three different combinations of STAPLE that are illustrated, whereas it was over-delineated when using *M*6 and *M*8 (Fig 1) or under-delineated by *M*5 and *M*7 (Fig 2).

#### Estimation of LVEF values for each method

The mean LVEF values and their standard deviations estimated for each subgroup of subjects are displayed in Table 1 for each initial segmentation method (*M*1 − *M*8) and each *MSi* method.

**Table 1:** Mean LVEF values (%) and their associated standard deviations.

Methods	HF-I (n = 12)	HF-NI (n = 12)	HYP (n = 12)	CTRL (n = 9)
*M*1	23.46±10.36	28.68±14.37	62.17±8.89	60.2±6.60
*M*2	25.12±10.55	31.93±14.20	65.39±6.35	66.18±4.98
*M*3	26.79±11.75	32.38±14.83	69.90±6.88	66.61±5.43
*M*4	24.15±11.75	33.30±16.94	64.95±12.02	66.51±6.07
*M*5	24.20±13.41	27.66±11.64	48.79±12.45	57.49±4.26
*M*6	25.81±13.19	35.04±17.71	73.94±10.62	74.30±6.73
*M*7	22.92±9.91	31.00±15.70	58.49±13.93	61.22±13.92
*M*8	31.47±13.13	35.95±15.19	69.50±10.19	68.22±10.86
*MS*45678	26.59±10.93	34.41±15.89	64.66±10.61	67.21±6.52
*MS*4567	24.23±10.44	33.42±14.84	61.75±11.37	65.36±5.86
*MS*4568	27.26±12.34	34.21±14.54	64.87±9.40	67.54±4.28
*MS*4578	27.01±12.23	32.97±14.71	59.15±11.79	64.20±5.18
*MS*4678	26.54±10.74	34.95±16.41	69.59±8.30	68.64±5.72
*MS*5678	26.26± 9.95	32.60±14.17	63.51±10.70	65.59±8.08
*MS*456	26.87±11.67	33.64±15.02	66.54±9.35	66.99±3.75
*MS*457	25.07±10.66	32.41±14.36	58.98±12.04	63.85±4.69
*MS*458	27.85±12.54	33.33±14.26	63.29±9.87	65.94±3.58
*MS*467	26.70±10.03	34.62±16.22	69.71±8.26	69.59±7.42
*MS*468	28.47±13.26	35.65±16.47	71.70±5.93	71.08±4.06
*MS*478	27.76±12.23	34.81±16.67	66.06±9.37	67.94±6.56
*MS*567	25.31±10.65	31.96±13.31	64.28±10.42	67.46±7.98
*MS*568	28.19±13.42	34.49±14.23	69.85±6.42	69.47±5.72
*MS*578	27.20±11.48	32.52±14.13	61.06±12.28	66.0±7.9
*MS*678	27.63±10.85	34.84±16.43	71.78±7.12	69.83±8.57

Values are computed for each segmentation method and for each subgroup of subjects: heart failure with and without ischemia patients (HF-I and HF-NI respectively), hypertrophic cardiomyopathy patients (HYP) and healthy individuals (CTRL).

### Supervised evaluation

#### Choice of the reference method

Table 2 presents the figures of merit computed using the eRWT approach when the eight initial segmentation methods (*M*1 − *M*8) were compared. These scores confirmed that *M*2 could be chosen as the reference method for the supervised evaluation. This result is similar to the one previously established in [3], despite the new values of the Beta distribution parameters. The performance of method *M*8 can be estimated by an improvement of its relative ranking.

**Table 2:** Figures of merit (*F*_*Mk*_) of the eight initial methods estimated by the eRWT approach.

Method	*M*1	*M*2	*M*3	*M*4	*M*5	*M*6	*M*7	*M*8
*F*_*Mk*_	0.003	< 0.001	0.001	0.004	0.015	0.008	0.010	0.008

#### Comparison of LVEF estimated values

**Fig 3:**
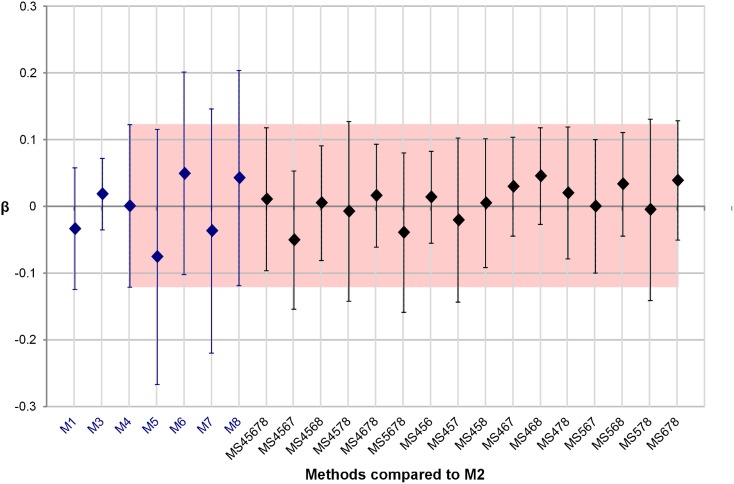
Supervised evaluation: Computation of the LVEF bias *β* of each method with respect to values obtained with *M*2 and its associated standard deviation. Error bars correspond to *β* ± 1.96*s*. The red box represents limits of agreement obtained for *M*4, the automated method whose results are closest to the *M*2 results for this evaluation.

Fig 3 shows the results obtained for the supervised evaluation. Each bias *β* with respect to the *M*2 result is represented with its associated standard deviations (error bars corresponding to ±1.96*s*). This figure shows that expert delineations *M*1 and *M*3 give the closest results to *M*2, with *M*3 showing less variability than *M*1. When comparing the five automated methods (*M*4 − *M*8), *M*4 yields the closest result to *M*2 with a bias near 0, and the smallest standard deviation (*s*). Although all semi-automated methods have slightly greater variability than the inter-expert variability, several STAPLE combinations are within the inter-expert variability, with six combinations presenting smaller variability than *M*1. Method *MS*456 was the one presenting the smallest variability [*β* ± 1.96*s*] among all *MSi*.

Among the sixteen tested *MSi* methods, ten were within the range [*β* ± 1.96*s*] obtained with *M*4. The six remaining *MSi* had a higher bias (in absolute value) than the one obtained with *M*4, but three of them (*MS*4567, *MS*5678 and *MS*678) had a lower *s* than *M*4. *MS*578 had a higher *s* than *M*4, but lower than the *s* obtained by the four methods used to create the STAPLE segmentation result. Finally, *MS*457 had a standard deviation *s* only 1% higher than the one obtained with *M*4, whereas *MS*4578 had a *s* 10% higher than the one obtained with *M*4.

### Unsupervised comparison of segmentation methods

Table 3 presents the ranking of the eight initial segmentation methods and of each STAPLE method *MSi*. Among the sixteen comparisons, method *MSi* was at a ranking similar to the experts in 14 cases (***bold and italic*** *MS* in the table). The best rank was reached by *MS*456 (rank equal to 2). Method *MS*578 was ranked like *M*4 (rank equal to 4, **bold** *MS* in the table), this rank being worse than the experts’ ranks but better than the individual methods used to create the combination. These results demonstrate that the LVEF parameters were more accurately estimated using this combination of segmentation methods than with any of the segmentation methods used in the combination. The worst rank observed for an *MSi* approach was obtained for *MS*4578 with a rank equal to 5 (*italic *MS** in the table), worse than *M*4 used to provide the STAPLE segmentation result. For this test, *F*_*M*1_ and *F*_*M*4_ were equal to 0.004, *F*_*MS*4578_ was equal to 0.005, and *F*_*M*8_ was equal to 0.007. So, even if *MS*4578 was at the fifth position, its figure of merit was close to the scores obtained with methods *M*1 and *M*4. Thus in this case, LVEF parameters estimated using *MSi* show a clear improvement compared to LVEF estimated using *M*5, *M*7 and *M*8.

**Table 3:** Ranking of the segmentation methods according to the different combinations of methods.

	Rank number	Methods entering the comparison with MS corresponding to:									
		MS45678	MS4567	MS4568	MS4578	MS4678	MS5678				
- Performance +	1	M2	M2-M3	M2	M2-M3	M2	M2				
	2	M3		M3		M3	M3				
	3	***MS***	***MS***	***MS***	M1-M4	***MS***	***MS***-M1				
	4	M1	M1-M4	M1-M4		M1-M4					
	5	M4			*MS*		M4				
	6	M8	M8-M6	M8-M6	M8	M8	M8				
	7	M6			M6	M6	M6				
	8	M7	M7	M7	M7	M7	M7				
	9	M5	M5	M5	M5	M5	M5				
	Rank number	Methods entering the comparison with MS corresponding to:									
		MS456	MS457	MS458	MS467	MS468	MS478	MS567	MS568	MS578	MS678
- Performance +	1	M2	M2	M2	M2	M2-M3	M2	M2	M2-M3	M2-M3	M2-M3
	2	M3-***MS***	M3	M3	M3		M3	M3			
	3		***MS***	***MS***	***MS***	***MS***	***MS***-M1	***MS***	***MS***	M1	***MS***
	4	M1-M4	M1-M4	M1-M4	M1-M4	M1-M4		M1	M1	M4-**MS**	M1
	5						M4	M4	M4		M4
	6	M8-M6	M8-M6	M8	M8-M6	M8	M8	M8-M6	M8	M8	M8
	7			M6		M6	M6		M6	M6	M6
	8	M7	M7	M7	M7	M7	M7	M7	M7	M7	M7
	9	M5	M5	M5	M5	M5	M5	M5	M5	M5	M5

***Bold and italic MS*** highlight methods MSi at an expert-like ranking. **Bold MS** highlights method MSi ranked behind the experts but in front of the individual methods used to create the combination. *Italic MS* highlights worst rank occupied by a method MSi.

## Discussion

### Use of STAPLE to combine endocardial LV segmentations

The aim of this work was to evaluate the efficiency of the STAPLE algorithm [5] to estimate a clinical biomarker, the LVEF, from a segmentation resulting from the combination of different independent segmentation algorithms. To demonstrate it, a collection of segmentations applied to the MICCAI 2009 cardiac MRI database was used. For the forty-five cases of this database eight segmentation methods were available, including delineations provided by three independent experts, and five delineations obtained using five automated LV segmentation algorithms. As the LVEF is a primordial biomarker, the paper primarily focused on results obtained for this parameter. The database had the advantage of including a large variety of cardiac diseases (with normal or reduced LVEF) and control subjects. The computation of the mean LVEF value and associated standard deviations for each subgroup showed that values were homogeneous for each subgroup of subjects, whatever the segmentation method used for the LVEF calculation. These first results confirmed that all segmentation methods provided coherent estimates for each subgroup of subjects.

Conventional applications of the STAPLE algorithm aim at defining a reference method from different expert segmentations [2, 5]. In the present study, our goal was not to define a consensus between “experts”, but rather to determine whether some combinations of different independent automated segmentation methods could yield a segmentation as reliable as that of an expert, keeping in mind that each automated method is slightly less powerful than expert delineation. In other words, could a combination of different automated segmentation results yield better results than the ones from each individual method? The question was challenging since several evaluation studies [2, 7] already showed that the STAPLE output strongly depends on the number and on the quality of the inputs used to create the combined segmentation. Assuming that the automated methods incorporate different strategies, we tested whether their combined use could actually help in improving segmentation results on a whole database. All possible combinations of three, four and five automated segmentations were thus systematically tested. As in [2], the STAPLE version that was used provided results that did not depend on the size of the background, i.e. the region of interest surrounding the left cavity. Furthermore STAPLE was tested on both 2D and 3D data, and better results were obtained when applying STAPLE in 2D mode, slice by slice. This seems to be due to the large anisotropy of the initial data and to the 2D strategy used by the experts and most of the automated algorithms to delineate the contours. To assess the segmentation results, a visual inspection of the contours of all STAPLE segmentation results superimposed onto the MR images was first performed. This visual assessment showed that in most cases, the STAPLE algorithm was able to correct, in every slice, too loose or too tight delineations obtained from automated methods. Supervised and unsupervised statistical evaluations were then performed to assess the results obtained using each STAPLE combination of three, four and five automated methods.

### Supervised evaluation

The main idea of the supervised evaluation was to compare the LVEF values estimated by all methods with the values computed by a “reference” method. We chose the *M*2 method as the “reference” method, as it yields the best figure of merit when using the eRWT approach on the eight initial methods. The comparison of LVEF values was based on the bias (*β*) and its associated standard deviation (*s*) obtained when computing LVEF values using each individual segmentation method compared to the *M*2 results (Fig 3). Furthermore, the combination of the three expert segmentation results using STAPLE, *MS*123, was estimated and chosen as the reference method. Results were very close to those obtained with the *M*2 method and the bias, as well as its associated standard deviation, were the smallest for method *M*2, confirming that this latter method was a good choice to be a reference method (see S1 Fig).

Results showed that among the five automated methods *M*4 was the closest to *M*2 with a low bias and the smallest standard deviation. In most cases, *MSi* results were closer to the reference method *M*2 than the original methods used in the combination, including *M*4 and were less variable than results obtained with each individual method. It can be concluded that the STAPLE algorithm provided segmentation results that yielded more accurate or equivalent results compared to the automated segmentation methods from which the STAPLE combination was based. Furthermore, the combination of three automated segmentation methods can provide a LVEF estimate as accurate as the one provided by an expert.

We noted that the bias related to each *MSi* method was correlated with the sum of the biases observed in the initial methods used in the combination (*r* = 0.736). We also observed a reduction of the standard deviation *s* when combining different methods using STAPLE, compared to the standard deviation of each individual method used in the STAPLE combination. However, the decrease in standard deviation was not directly predictable.

### Ranking provided by the eRWT approach

The eRWT approach ranked the expert delineation *M*2 first, and more generally, the three expert delineations in the top three. The semi-automated method *M*4 was ranked as the best automated method to estimate LVEF.

To evaluate the STAPLE segmentation results (*MSi*) without using strong *a priori* on the truth, the eRWT approach was systematically applied to the eight original methods and to an *MSi* method. In most cases, *MSi* ranked similarly to the expert delineations (*M*3 and *M*1). This means that the STAPLE algorithm based on several automated methods provided similar results to those obtained by experts. In one case (*MS*578), the rank of the STAPLE method was less than those of experts but was still better than those of the three methods STAPLE was based on. This suggests that the LVEF parameters were once again better estimated using the combination of segmentation methods than using any of each initial segmentation method used in STAPLE. Finally, in only one instance (*MS*4578), *MSi* was ranked after one of the four methods (*M*4) used in the combination. However, the figures of merit showed that LVEF parameters estimated using *MSi* were better than those estimated using three of the four methods involved in the combination (*M*5, *M*7 and *M*8). Furthermore, results obtained with (*MS*4578) were very close to those obtained with *M*4.

Furthermore, both supervised and unsupervised statistical approaches led to very similar conclusions. Indeed, both approaches showed that the most accurate LVEF was obtained when combining *M*4, *M*5, and *M*6. Furthermore, both approaches showed that the poorest results were obtained when combining *M*4, *M*5, *M*7 and *M*8. This *a posteriori* consistency between conclusions suggests that the use of the unsupervised eRWT approach was relevant in our context and that the different hypotheses underlying the eRWT approach proved to be realistic.

### Extension to other cardiac function parameters

The present work mainly focused on the estimation of the LVEF value. As this parameter is derived from both end-diastolic volumes (*EDV*) and end-systolic volumes (*ESV*), additional tests have been performed to extend our study to five other clinical parameters: the left ventricular end-diastolic volumes and end-systolic volumes, the stroke volume (*SV* = *EDV* − *ESV*), the epicardial volume (*EpV*) defined at the end-diastolic phase, and the myocardial mass (*MM* = 1.05 × (*EpV* − *EDV*)). Epicardial borders, obtained on the end-diastolic phases for all methods except *M*7, and used to study the *EpV* and the *MM* parameters, are also available on https://github.com/frederiquefrouin/Medieval.

The eRWT approach was performed for each clinical parameter with specific settings (see S1 Table). Results shown in S2 Table confirmed that: 1) method *M*2 was the best “reference” method, 2) the three expert delineations were ranked among the top three methods, and 3) the semi-automated method M4 was the best automated method to estimate any clinical parameter. S2 Table also showed a limitation for the study of *ESV* for which the ranking of the eight original methods was quite different from the expected results, ranking for instance method *M*7 first. A detailed analysis revealed the presence of an outlier with an *ESV* of about 430 ml (estimated by the experts), while this *ESV* was less than 310 ml for all other subjects. When removing this outlier, the ranking became similar as for the other cases, *i.e.* with experts methods ranked first.

The supervised evaluation based on the computation of the bias *β* and its associated standard deviation (*s*) for each segmentation method and each STAPLE combination with respect to the reference method *M*2 was performed for these five supplementary clinical parameters (S2–S6 Figs). Our results confirmed that all clinical parameters were better estimated when combining segmentation methods with STAPLE than when using one of the individual methods entering the STAPLE combination.

Tests performed on the estimation of LVEF showed that the combination of *M*4, *M*5 and *M*6 provided the best estimate (Table 3). To further investigate this result, we applied the eRWT approach to the different combinations of three automated segmentation methods (S3–S4 Tables). Results suggest that method *MS*458 is an appropriate combination, since it ranked first or second for all clinical parameters except for the end-diastolic volume for which it ranked in the third group of methods.

The major interest of the eRWT approach that provides a ranking of different estimation methods based on only few *a priori* hypotheses is underlined here as its results appeared robust for different clinical parameters described by a large variety of statistical distribution (S1 Table).

### Limitations

As underlined in the Material and Methods section, our study focused on the left ventricle segmentation. For that reason, we did not study the impact of the choice of end-diastolic and end-systolic phases in the variability of clinical parameters. Preliminary investigations were performed using the segmentation results of all cardiac phases provided by method *M*5. They showed that choosing the systolic and diastolic phases as the phases providing the smallest and largest volumes respectively was not the largest source of variability in the LVEF estimation. The selection of the more basal and the more apical slices to segment is another source of variability. For that point, we strongly believe that a minimal intervention of the user could help the automated algorithms, without being time consuming.

### Future directions

The statistical tools that were used for this study could also be used to compare the STAPLE algorithm with other algorithms that have been developed to define representative contours from a collection of contours; it could be for instance, the ones described in [7] or in [8]. This could help identify the most efficient algorithm to combine contours. However, this would require testing the statistical independency of *σ*_*k*_ in the eRWT model (Eq (1)) when comparing different methods of combination based on the same initial methods.

Due to the difficulty in getting one or multiple expert delineations for clinical segmentation problems, the combined use of different independent algorithms could yield a valuable alternative. Of course, the combination process requires some computing resources, which depend on the segmentation methods involved in the combination and on the method used for combining them (here STAPLE) but it guarantees reproducible results and manual delineation is no longer needed. Due to the quality of results demonstrated by this study, which shows a clear improvement in clinical parameter estimates using the STAPLE combinations compared to the initial automated segmentation algorithms, it becomes feasible to use automated segmentation algorithms and get stable and reliable results.

Finally our approach was dedicated to LV segmentation, but it could be easily extended to other organs. One practical interest of such an approach is that it could help in reducing the number of manual delineations which are very time-consuming to collect, especially for databases including a large number of cases. Furthermore, we believe that the high performance obtained by the combination is due to the complementarity of the different segmentation approaches and that results could be less interesting when tuning parameters of a single approach. However this latter hypothesis remains to be fully evaluated.

## Conclusion

This work aimed at determining whether combining different segmentation results using the STAPLE algorithm could yield a final segmentation as reliable as that of an expert. This approach was tested in the framework of the estimation of left ventricular ejection fraction on the MICCAI 2009 cardiac cine MRI database. Both supervised and unsupervised evaluations showed that in most cases, the cardiac function parameters were better estimated using the STAPLE approach than using individually the segmentation methods used to create the STAPLE result. Moreover, the STAPLE segmentation results provided, in most cases, similar estimates to the ones obtained based on manual delineations performed by an expert. The results show that combining different independent automated segmentation methods using the STAPLE approach yielded segmentations that were as accurate as those provided by expert delineating the left ventricular cavities.

## Supporting Information

S1 Fig Comparison of *LVFE* estimated by the different methods with *LVFE* provided by method *MS*123. (PDF)

S2 Fig Comparison of *EDV* estimated by the different methods with *EDV* provided by method *M*2. (PDF)

S3 Fig Comparison of *ESV* estimated by the different methods with *ESV* provided by method *M*2. (PDF)

S4 Fig Comparison of *SV* estimated by the different methods with *SV* provided by method *M*2. (PDF)

S5 Fig Comparison of *EpV* estimated by the different methods with *EpV* provided by method *M*2. (PDF)

S6 Fig Comparison of *MM* estimated by the different methods with *MM* provided by method *M*2. (PDF)

S1 Table Range of values and setting of eRWT for the different clinical parameters. (PDF)

S2 Table Ranking of the eight original methods provided by eRWT for the different clinical parameters. (PDF)

S3 Table Ranking of the ten combinations of three automated methods (among 5) using STAPLE for endocardial based indices. (PDF)

S4 Table Ranking of all the combinations of automated methods using STAPLE for epicardial based indices. (PDF)

## References

[1] Petitjean C. and Dacher J.-N., “A review of segmentation methods in short axis cardiac MR images.,” *Med Image Anal*, vol. 15, pp. 169–184, 4 2011 10.1016/j.media.2010.12.004 21216179

[2] Suinesiaputra A., Cowan B. R., Al-Agamy A. O., Elattar M. A., Ayache N., Fahmy A. S., et al, “A collaborative resource to build consensus for automated left ventricular segmentation of cardiac MR images.,” *Med Image Anal*, vol. 18, pp. 50–62, 1 2014 10.1016/j.media.2013.09.001 24091241 PMC3840080

[3] Lebenberg J., Buvat I., Lalande A., Clarysse P., Casta C., Cochet A., et al, “Nonsupervised ranking of different segmentation approaches: application to the estimation of the left ventricular ejection fraction from cardiac cine MRI sequences.,” *IEEE Trans Med Imaging*, vol. 31, pp. 1651–1660, 8 2012 10.1109/TMI.2012.2201737 22665506

[4] Chalana V. and Kim Y., “A methodology for evaluation of boundary detection algorithms on medical images,” *IEEE Trans Med Imaging*, vol. 16, no. 5, pp. 642–652, 1997 10.1109/42.640755 9368120

[5] Warfield S. K., Zou K. H., and Wells W. M., “Simultaneous truth and performance level estimation (STAPLE): an algorithm for the validation of image segmentation,” *IEEE Trans Med Imaging*, vol. 23, pp. 903–921, 7 2004 10.1109/TMI.2004.828354 15250643 PMC1283110

[6] Chen A., Niermann K. J., Deeley M. A., and Dawant B. M., “Evaluation of multiple-atlas-based strategies for segmentation of the thyroid gland in head and neck ct images for imrt.,” *Phys Med Biol*, vol. 57, pp. 93–111, 1 2012 10.1088/0031-9155/57/1/93 22126838 PMC3505993

[7] Robitaille N. and Duchesne S., “Label fusion strategy selection.,” *Int J Biomed Imaging*, vol. 2012, p. 431095, 2012 10.1155/2012/431095 22518113 PMC3296312

[8] S. Jehan-Besson, C. Tilmant, A. De Cesare, A. Lalande, A. Cochet, J. Cousty, et al., “A mutual reference shape based on information theory.,” in *Conf Proc IEEE International Conference on Image Processing*, (Paris, France), pp. 887–891, Oct 2014.

[9] Dice L., “Measures of the amount of ecologic association between species,” *Ecology*, vol. 26, pp. 297–302, 7 1945 10.2307/1932409

[10] Radau P., Lu Y., Connelly K., Paul G., Dick A., and Wright G., “Evaluation framework for algorithms segmenting short axis cardiac MRI,” in *The MIDAS Journal—Cardiac MR Left Ventricle Segmentation Challenge*, 2009 Available: http://hdl.handle.net/10380/3070.

[11] Constantinides C., Chenoune Y., Kachenoura N., Roullot E., Mousseaux E., Herment A., et al, “Semi-automated cardiac segmentation on cine magnetic resonance images using GVF-Snake deformable models,” in *The MIDAS Journal—Cardiac MR Left Ventricle Segmentation Challenge*, 2009 Available: http://hdl.handle.net/10380/3108.

[12] Schaerer J., Casta C., Pousin J., and Clarysse P., “A dynamic elastic model for segmentation and tracking of the heart in MR image sequences,” *Med Image Anal*, vol. 14, pp. 738–749, 12 2010 10.1016/j.media.2010.05.009 20598934

[13] Cousty J., Najman L., Couprie M., Clement-Guinaudeau S., Goissen T., and Garot J., “Segmentation of 4D cardiac MRI: Automated method based on spatio-temporal watershed cuts,” *Image Vision Comput*, vol. 28, pp. 1229–1243, 8 2010 10.1016/j.imavis.2010.01.001

[14] Lalande A., Salvé N., Comte A., Jaulent M. C., Legrand L., Walker P. M., et al, “Left ventricular ejection fraction calculation from automatically selected and processed diastolic and systolic frames in short-axis cine-MRI,” *J Cardiovasc Magn Reson*, vol. 6, pp. 817–827, 2004 10.1081/JCMR-200036143 15646885

[15] C. Constantinides, E. Roullot, M. Lefort, and F. Frouin, “Fully automated segmentation of the left ventricle applied to cine mr images: description and results on a database of 45 subjects.,” *Conf Proc IEEE Eng Med Biol Soc*, vol. 2012, pp. 3207–3210, 2012. 10.1109/EMBC.2012.6346647 23366608

[16] Constantinides C., Chenoune Y., Mousseaux E., Frouin F., and Roullot E., “Automated heart localization for the segmentation of the ventricular cavities on cine magnetic resonance images,” in *Computing in Cardiology*, vol. 37, (Belfast, Ireland), pp. 911–914, 9 26–29 2010.

[17] Dempster A. P., Laird N. M., and Rdin D. B., “Maximum Likelihood from Incomplete Data via the EM Algorithm,” in *Journal of Th Royal Statistical Society, series B*, vol. 39, pp. 1–38, 1977.

[18] McLachlan G. J. and Krishnan T., *The EM Algorithm and Extensions*. Wiley-Interscience, 1 ed., 11 1996.

[19] Kupinski M. A., Hoppin J. W., Krasnow J., Dahlberg S., Leppo J. A., King M. A., et al, “Comparing cardiac ejection fraction estimation algorithms without a gold standard,” *Acad Radiol*, vol. 13, pp. 329–337, 3 2006 10.1016/j.acra.2005.12.005 16488845 PMC2464280

[20] Hoppin J. W., Kupinski M. A., Kastis G. A., Clarkson E., and Barrett H. H., “Objective comparison of quantitative imaging modalities without the use of a gold standard,” *IEEE Trans Med Imaging*, vol. 21, pp. 441–449, 5 2002 10.1109/TMI.2002.1009380 12071615 PMC3150581

[21] Kupinski M. A., Hoppin J. W., Clarkson E., Barrett H. H., and Kastis G. A., “Estimation in medical imaging without a gold standard,” *Acad Radiol*, vol. 9, pp. 290–297, 3 2002. 11887945 10.1016/s1076-6332(03)80372-0 PMC3143018

[22] Soret M., Alaoui J., Koulibaly P. M., Darcourt J., and Buvat I., “Accuracy of partial volume effect correction in clinical molecular imaging of dopamine transporter using SPECT,” *Nuclear Instruments and Methods in Physics Research A*, vol. 571, pp. 173–176, 2 2007 10.1016/j.nima.2006.10.236

[23] Efron B. and Tibshirani R. J., *An Introduction to the Bootstrap*. New York: Chapman & Hall, 1993.

[24] Kruskal W. H. and Wallis W. A., “Use of Ranks in One-Criterion Variance Analysis,” *Journal of the American Statistical Association*, vol. 47, pp. 583–621, 12 1952 10.1080/01621459.1952.10483441

[25] Miller R. G., *Simultaneous Statistical Inference*. New York: Springer Verlag, 2nd ed., 1981.

