# Supplementary material for: Improved Estimation of Cardiac Function Parameters Using a Combination of Independent Automated Segmentation Results in Cardiovascular Magnetic Resonance Imaging
Source: PLoS One. 2015 Aug 19;10(8):e0135715. doi: 10.1371/journal.pone.0135715 (PMC4545395; doi:10.1371/journal.pone.0135715)
Supplement: S1 Fig — (PDF) [file pone.0135715.s001.pdf]

S 1. Fig. Comparison of $LVFE$ estimated by the different methods with $LVFE$ provided by method $MS123$.

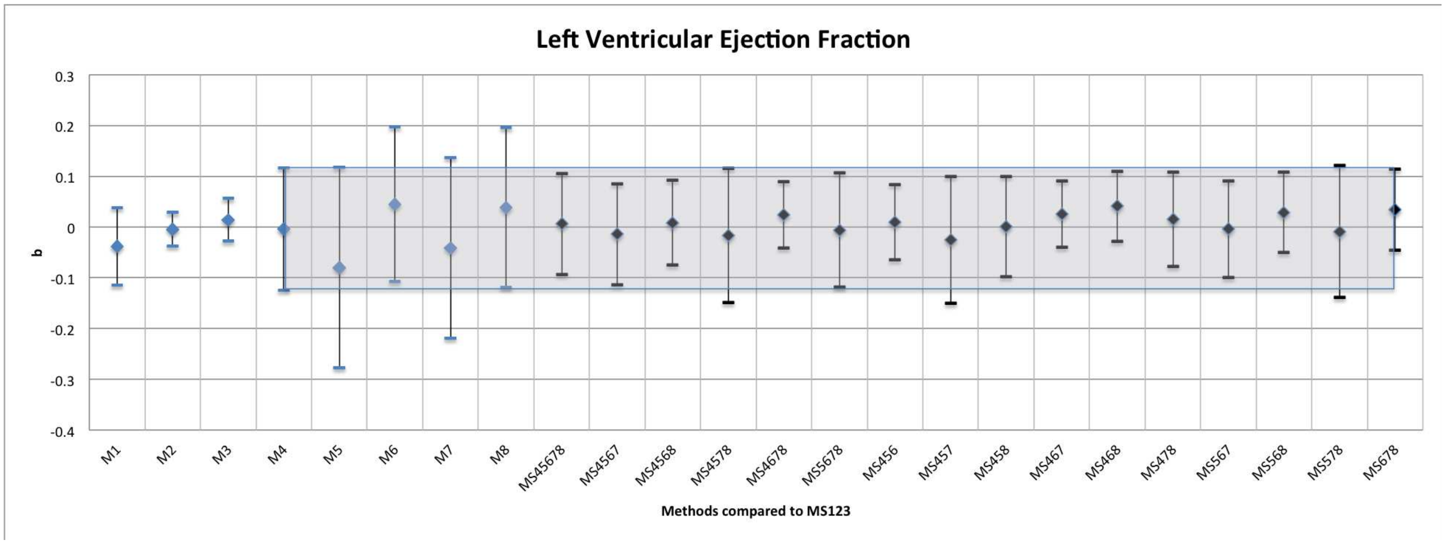

Each diamond represents the bias β of the method with respect to values obtained with $MS123$ and error bars correspond to limits of agreement $\beta \pm 1.96s$.
