# Supplementary material for: Improved Estimation of Cardiac Function Parameters Using a Combination of Independent Automated Segmentation Results in Cardiovascular Magnetic Resonance Imaging
Source: PLoS One. 2015 Aug 19;10(8):e0135715. doi: 10.1371/journal.pone.0135715 (PMC4545395; doi:10.1371/journal.pone.0135715)
Supplement: S4 Fig — (PDF) [file pone.0135715.s004.pdf]

S 4. Fig. Comparison of SV estimated by the different methods with SV provided by method $M2$.

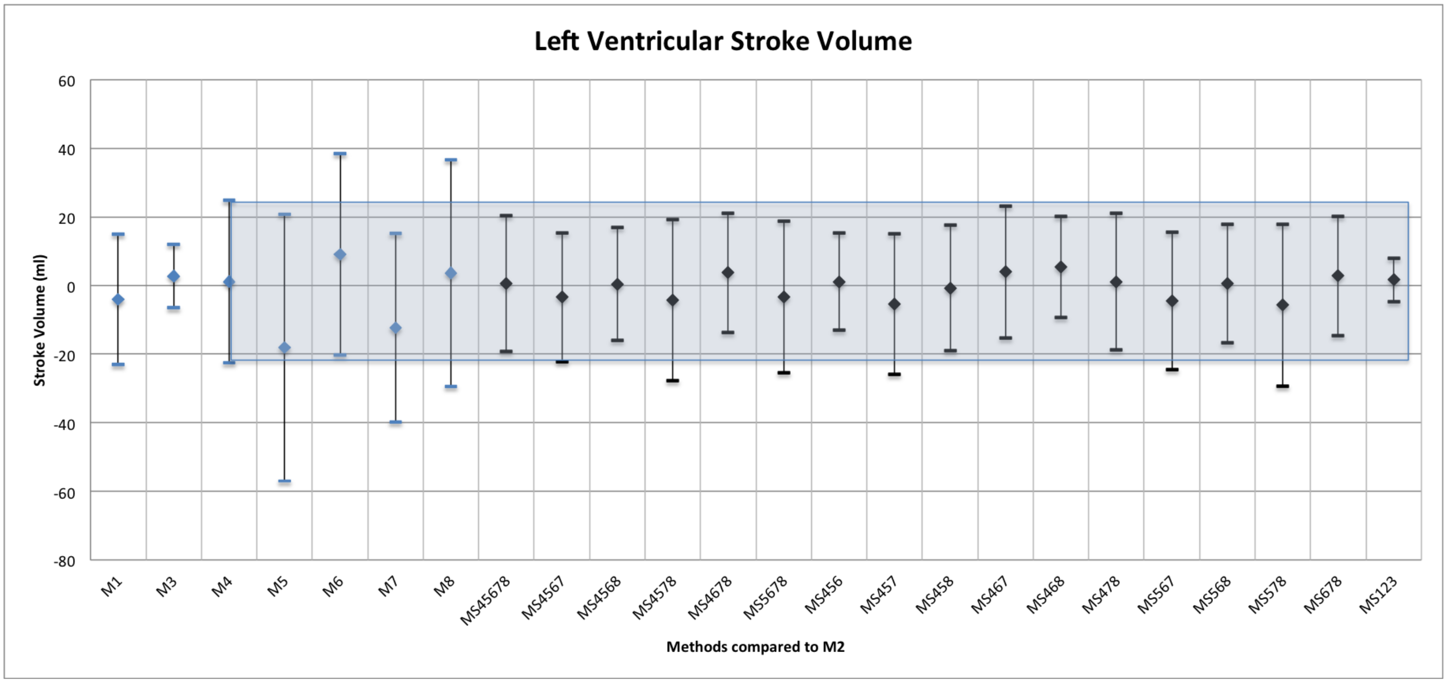

Each diamond represents the bias β of the method with respect to values obtained with $M2$ and error bars correspond to limits of agreement $\beta \pm 1.96s$.
