# Supplementary material for: Improved Estimation of Cardiac Function Parameters Using a Combination of Independent Automated Segmentation Results in Cardiovascular Magnetic Resonance Imaging
Source: PLoS One. 2015 Aug 19;10(8):e0135715. doi: 10.1371/journal.pone.0135715 (PMC4545395; doi:10.1371/journal.pone.0135715)
Supplement: S1 Table — (PDF) [file pone.0135715.s007.pdf]

S 1. Table. Range of values and setting of eRWT for the different clinical parameters.

Parameters	Lower Bound	Upper Bound	μ	ν
Ejection Fraction ($LVEF$)	0	1	2.85	3.4
End-Diastolic Volume (EDV)	30 ml	505 ml	2.5	5.5
End-Systolic Volume (ESV)	0 ml	450 ml	2	5.75
End-Systolic Volume* (ESV^*)	0 ml	305 ml	2.85	3.15
Stroke Volume (SV)	10 ml	165 ml	3.15	5.25
Epicardial Volume (EpV)	75 ml	750 ml	2.5	2.25
Myocardial Mass (MM)	25 g	360 g	2.5	5.6

ESV^* is defined by removing one subject from the population, this subject presenting a very large end systolic volume.
