# Supplementary material for: Improved Estimation of Cardiac Function Parameters Using a Combination of Independent Automated Segmentation Results in Cardiovascular Magnetic Resonance Imaging
Source: PLoS One. 2015 Aug 19;10(8):e0135715. doi: 10.1371/journal.pone.0135715 (PMC4545395; doi:10.1371/journal.pone.0135715)
Supplement: S2 Table — (PDF) [file pone.0135715.s008.pdf]

S 2. Table. Ranking of the eight original methods provided by eRWT for the different clinical parameters.

	Rank number	$LVEF$	EDV	ESV	ESV^*	SV	EpV	MM
- Performance +	1	$M2$	$M3 - M1 - M2 - M4$	$M7$	$M1$	$M2$	$M2$	$M2$
	2	$M3$		$M2$	$M2$	$M3$	$M1$	$M1$
	3	$M1$		$M3 - M8 - M4$	$M4 - M3$	$M1 - M4$	$M3$	$M3$
	4	$M4$					$M4$	$M4$
	5	$M6 - M8$	$M6$		$M7$	$M6 - M8$	$M5$	$M5$
	6		$M8$	$M1 - M5$	$M5$		$M6 - M8$	$M8 - M6$
	7	$M7$	$M7$		$M6$	$M7$		
	8	$M5$	$M5$	$M6$	$M8$	$M5$		

ESV^* is defined by removing one subject from the population, this subject presenting a very large end systolic volume.
