# Supplementary material for: Improved Estimation of Cardiac Function Parameters Using a Combination of Independent Automated Segmentation Results in Cardiovascular Magnetic Resonance Imaging
Source: PLoS One. 2015 Aug 19;10(8):e0135715. doi: 10.1371/journal.pone.0135715 (PMC4545395; doi:10.1371/journal.pone.0135715)
Supplement: S3 Table — (PDF) [file pone.0135715.s009.pdf]

S 3. Table. Ranking of the ten combinations of three automated methods (among 5) using STAPLE for endocardial based indices.

	Rank number	$LVEF$	EDV	ESV
- Performance +	1	$MS456$–**MS458**–$MS478$	$MS578$	$MS578$
	2		$MS567 - MS457 - MS568^{\#}$	$MS457$–**MS458**–$MS478$
	3			
	4	$MS467$	$MS568^{\#} - MS478$–**MS458**–$MS678$	
	5	$MS568$		$MS567 - MS568 - MS678$
	6	$MS567 - MS678 - MS468$		
	7			
	8		$MS468 - MS456 - MS467$	$MS467 - MS456 - MS468$
	9	$MS578$		
	10	$MS457$		

	Rank number	ESV^*	SV
- Performance +	1	$MS457$	$MS478$
	2	**MS458**–$MS456^{\#}$	$MS468 - MS568-$ **MS458**
	3	$MS456^{\#} - MS467 - MS478 - MS567 - MS578$	
	4		
	5		$MS678 - MS456$
	6		
	7		$MS467$
	8	$MS678 - MS468$	$MS457 - MS567^{\#}$
	9		$MS567^{\#} - MS578$
	10	$MS568$	

ESV^* is defined by removing one subject from the population, this subject presenting a very large end systolic volume. $^{\#}$ represents methods belonging to two consecutive groups.
