# Supplementary material for: Improved Estimation of Cardiac Function Parameters Using a Combination of Independent Automated Segmentation Results in Cardiovascular Magnetic Resonance Imaging
Source: PLoS One. 2015 Aug 19;10(8):e0135715. doi: 10.1371/journal.pone.0135715 (PMC4545395; doi:10.1371/journal.pone.0135715)
Supplement: S4 Table — (PDF) [file pone.0135715.s010.pdf]

S 4. Table. Ranking of all the combinations of automated methods using STAPLE for epicardial based indices.

	Rank number	EpV	MM
- Performance +	1	**MS458**	$MS468$
	2	$MS468$	**MS458**$-MS4568$
	3	$MS568 - MS4568$	
	4		$MS456$
	5	$MS456$	$MS568$
